# High-fidelity, personalized cardiac modeling via AI-driven 3D reconstruction and embedded silicone rubber printing

**DOI:** 10.3389/ebm.2025.10756

**Published:** 2025-10-01

**Authors:** Xuefang Wang, Yixin Li, Zhiqi Liang, Ruxu Du, Ting Song

**Affiliations:** ^1^ Shien-Ming Wu School of Intelligent Engineering, South China University of Technology, Guangzhou, China; ^2^ Department of Radiology, Guangdong Provincial Key Laboratory of Major Obstetric Diseases, Guangdong Provincial Clinical Research Center for Obstetrics and Gynecology, The Third Affiliated Hospital, Guangzhou Medical University, Guangzhou, China; ^3^ Guangzhou Janus Biotechnology Co., Ltd., Guangzhou, China

**Keywords:** cardiac 3D reconstruction, embedded 3D printing, cardiac segmentation, silicone rubber matrix, deep learning

## Abstract

The burgeoning clinical demand for patient-specific cardiac modeling encounters significant challenges. The current clinical cardiac models are either difficult to manufacture or lack of detailed geometric structures and hence, often fail to incorporate important patient-specific characteristics. Moreover, most 3D-printable soft materials, such as Thermoplastic Poly-Urethane (TPU) or elastic resins, exhibit insufficient flexibility and biocompatibility to accurately mimic cardiac tissues, therefore limiting their ability to truly replicate patient-specific cardiac conditions. To address these limitations, we propose an innovative method for patient-specific cardiac substructure reconstruction based on the integration of Artificial Intelligence (AI) and embedded 3D printing. First, by combining medical imaging data (CT scan) with AI-driven high-precision 3D reconstruction algorithms, the new method segments the patient-specific cardiac structure into 10 substructures. The average Dice coefficient across the ten substructures is 0.87. Second, it uses an embedded 3D printing technique which utilizes silicone rubber matrix as supporting structure and uses diluted catalyst ink to extrude onto the supporting matrix. Through precise regulation of the matrix composition, material deposition rate and curing time, it can fabricate high-fidelity, complex 3D patient-specific silicone heart models with the average dimensional error less than 0.5 mm. The proposed method can substantially reduce manual intervention and post-processing time. The fabricated models provide valuable morphological insights for cardiovascular diagnosis and treatment planning. It is believed that many clinic applications will follow.

## Impact statement

We propose a novel framework integrating artificial intelligence (AI) and embedded 3D printing for personalized cardiac substructure reconstruction through two primary contributions: High-precision 3D reconstruction algorithm: Utilizing medical imaging data (CT scans), we developed a cardiac substructure segmentation framework incorporating anatomical priors with correlated spatial-channel co-attention mechanisms. This system enables automatic identification and precise segmentation of cardiac substructures, significantly enhancing image resolution and data fidelity during the 3D printing preprocessing stage, thereby generating high-quality 3D printable files. Embedded 3D printing of silicone rubber matrix: By precisely modulating the composition ratio of base materials within the supporting matrix, we achieved on-demand printing-curing synchronization. This innovative approach effectively addresses the rheological challenges associated with pre-cured silicone, enabling the fabrication of complex three-dimensional cardiac models with exceptional anatomical fidelity. This approach yields high-fidelity 3D silicone cardiac models (dimensional error <0.5 mm), accurately replicating patient-specific anatomy to support precise diagnosis and treatment planning.

## Introduction

Cardiovascular diseases (CVDs) represent 33% of worldwide deaths (20.5 million/year), with persistently rising mortality rates (WHF [1]). Effective treatment requires not only targeted interventions but also personalized medical care. Developing accurate human heart models is crucial for understanding cardiac pathology and guiding diagnosis and treatment. The heart is an intricately complex and multifunctional organ, encompassing a sophisticated vascular network and atrial and ventricular structures. Accurately replicating the complex structures of the heart remains a significant challenge, primarily manifested in three critical aspects. First, the initial and most crucial step involves utilizing high-resolution medical imaging technologies combined with advanced algorithms to precisely capture the intricate details of cardiac structures. Second, the selection of an appropriate 3D printing technology is essential, as it must provide high resolution and detail fidelity to ensure the accuracy of the models. Third, identifying suitable printing materials that can mimic the softness and elasticity of the heart while maintaining sufficient strength and stability during the fabrication process is vital.

High-resolution imaging and advanced algorithms form the foundation for precise cardiac modeling, with substructure segmentation remaining a core research focus. While deep learning has revolutionized this field through automated feature extraction and nonlinear modeling [2–9], current approaches still face significant challenges: (1) The U-Net model proposed by Ronneberger et al. [10], a convolutional neural network architecture for biomedical image segmentation, achieved groundbreaking results in medical image segmentation. However, U-Net-based frameworks [11, 12] and their variants (e.g., dense U-Net for multi-scale features [13]): often struggle with cardiac substructures’ scale variations and topological complexity. (2) Most methods neglect inherent cardiac anatomy, reducing effectiveness. (3) Many algorithms require high-performance hardware and extensive labeled data, leading to poor performance on fine substructures. Despite multi-stage solutions [14–16] that decompose segmentation into region-of-interest (ROI) localization and refinement, significant gaps remain in precision and robustness for clinical application.

Recent advances in 3D-printed cardiac models show promise for improving cardiac care, yet challenges remain. While integration of deep learning has enhanced segmentation accuracy (e.g., coronary artery identification [17]) and streamlined clinical workflows [18], current approaches often lack comprehensive solutions. Three major limitations persist: (1) manufacturing complexity and limited geometric fidelity hinder high-precision applications [19–21]; (2) commercially available soft materials (e.g., TPU, elastic resins); fail to adequately replicate cardiac tissue properties; (3) conventional 3D printing struggles with soft materials due to flowability, support requirements, and precision constraints.

In conclusion, the current limitations of existing models fail to meet the essential requirements for personalized and accurate replication of cardiac structures. To address these challenges, this study focuses on two dimensions: (1) enhancing data accuracy during the pre-processing stage of 3D printing to improve the precision of cardiac substructure replication; (2) exploring the optimal printing technologies and materials that can simultaneously mimic the soft elasticity of cardiac tissue while ensuring structural integrity during the manufacturing process. We propose a new solution leveraging advanced imaging data (e.g., CT scans) and high-precision 3D reconstruction algorithms to automatically identify and segment cardiac substructures from medical imaging data, thereby generating high-quality 3D printable files. By harnessing the capabilities of embedded 3D printing technology, we have engineered a specialized 3D printing system for fabricating silicone rubber-based constructs. This system enables the deposition of soft materials within a temporary support matrix, which is subsequently removed to yield intricate, patient-specific structures. This methodology simultaneously addresses challenges related to material flowability, support structure requirements, and printing precision, thereby enabling the production of highly customized cardiac models.

These patient-specific silicone cardiac models are designed for direct clinical translation. As tangible, anatomically accurate replicas, they facilitate the understanding of cardiac pathology, aid in diagnostic decision-making, and support preoperative planning by allowing surgeons to visualize spatial anatomical relationships, assess procedural feasibility, and simulate surgical interventions (e.g., resection, implantation, or repair) prior to actual surgery. Additionally, the models provide a realistic tactile experience for training residents and fellows in cardiac anatomy and interventional techniques, reducing reliance on cadavers and live procedures. By bridging imaging, engineering, and clinical practice, this platform can enhances training efficacy while offering support for the development of personalized therapeutic strategies.

## Methodology

**FIGURE 1:**
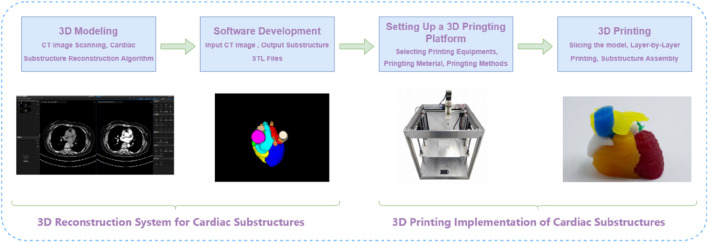
The integrated framework for 3D reconstruction of cardiac substructures.

To achieve precise reconstruction of the cardiac anatomical structure, we propose an integrated framework for 3D reconstruction of cardiac substructures, comprising two core subsystems: a 3D reconstruction system for cardiac substructures and an embedded 3D printing system for silicone rubber-based constructs, as depicted in Figure 1. This framework systematically addresses multiple critical stages, including the 3D modeling of ten cardiac substructures derived from medical CT images using advanced reconstruction algorithms, the development of specialized 3D reconstruction software, and the design and implementation of optimized 3D printing materials and a robust printing platform.

### Three-dimensional reconstruction system for cardiac substructures

The three-dimensional (3D) reconstruction system for cardiac substructures was developed based on a proprietary algorithm specifically designed for cardiac imaging. This integrated system streamlines the entire workflow from cardiac computed tomography (CT) image acquisition to the generation of stereolithography (STL) files suitable for 3D printing applications, enabling automated and intelligent reconstruction of cardiac substructures.

#### High-precision reconstruction algorithm for cardiac substructures

**FIGURE 2:**
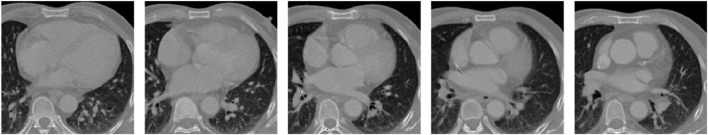
CT slice sequence of cardiac substructures.

Cardiac substructures present several technical challenges in CT imaging, including heterogeneous grayscale intensities, poorly defined boundaries, irregular morphological features, and positional variability. Adjacent substructures often have similar grayscale values, resulting in low contrast, while some are connected via blood inflow pathways and differ significantly in size. Moreover, cardiac morphology and spatial orientation vary considerably across individuals, and even within the same subject over time or across imaging planes (as illustrated in Figure 2). Due to the inherent limitations of medical imaging modalities and tissue-specific properties, images are susceptible to artifacts such as noise and motion-induced distortions. These intrinsic challenges pose significant difficulties for accurate reconstruction of cardiac substructures.

The experimental data were acquired from the Third Affiliated Hospital of Guangzhou Medical University, with prior approval from the Institutional Review Board (IRB). Cardiac CT imaging data from 117 clinical cases were collected for this study. Non-contrast CT scans were used in this study. All included cases had normal cardiac anatomy, with no significant pathological conditions or anatomical variants. Cases were selected via random sampling from the institutional database to ensure representativeness and minimize selection bias. These cases include precise contour annotations of ten key cardiac structures: the left and right atria (LA and RA), left and right ventricles (LV and RV), superior and inferior vena cava (SVC and IVC), pulmonary artery (PA), pulmonary vein (PV), and ascending and descending aorta (AA and DA). The annotation task was collaboratively performed by three experienced radiologists, with two intermediate-level physicians performing the initial delineation, which was subsequently reviewed and confirmed by a senior physician.

**FIGURE 3:**
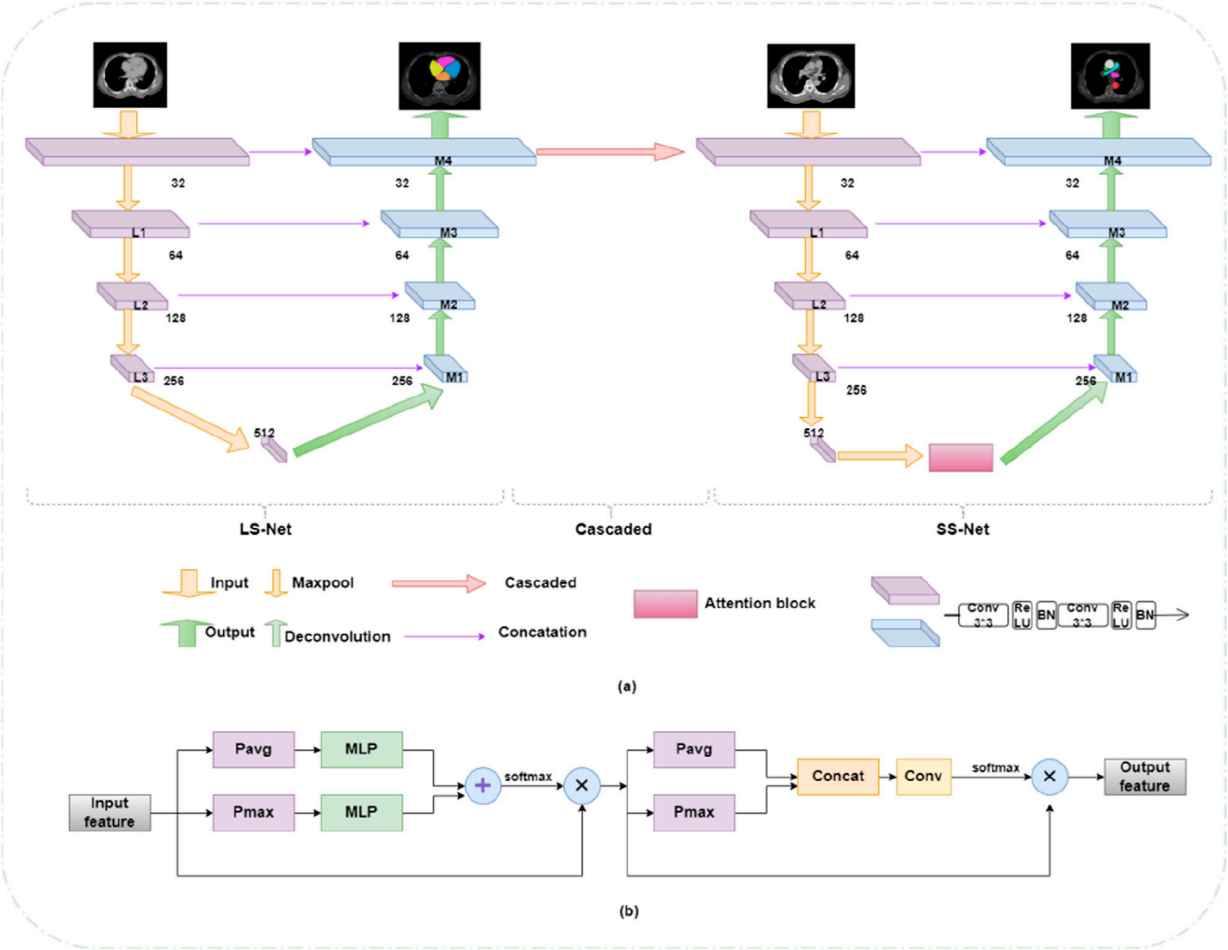
**(a)** Cardiac substructure segmentation framework integrating anatomical priors with collaborative spatial-channel attention mechanisms. **(b)** Collaborative attention module.

In addressing the challenges associated with reconstructing substructures in cardiac CT images and the inherent limitations of current deep learning segmentation models, our approach is inspired by the meticulous delineation techniques employed by medical professionals. We have developed a cardiac substructure segmentation framework that synergistically combines anatomical prior knowledge with a spatial-channel co-attention mechanism (as depicted in Figure 3). This architecture represents an advanced iteration of a segmentation framework predicated on anatomical structure priors, as delineated in the seminal work [22]. This framework employs a sequential two-step segmentation process, utilizing a coarse-to-fine cascade network. The initial step involves coarse segmentation (Large substructure segmentation network LS-Net, depicted on the left side of Figure 3a) of more readily identifiable substructures (such as the quartet of atrial and ventricular formations), followed by fine segmentation (Small substructure segmentation network SS-Net, illustrated on the right side of Figure 3a) of more complex substructures (such as the quartet of atrial and ventricular formations). To augment the segmentation accuracy of small-scale substructures, the outcomes of the coarse segmentation are utilized as prior information, which, in conjunction with the original image, constitutes the model’s input. The anatomical knowledge pertaining to large-scale substructures is embedded within the fine segmentation network to guide and refine the training of small-scale substructures. At the interface between the encoder and decoder in the fine segmentation network, a spatial-channel co-attention module is strategically designed to adaptively compute the region of interest based on the channel and spatial distribution information of the features themselves (illustrated in pink block in Figure 3a, with detailed architecture shown in Figure 3b). This module adeptly extracts spatial and channel data from feature maps across diverse scales, assimilates long-range dependencies among features of varying magnitudes, and computes the weighting of target features across multiple dimensions. Consequently, it captures the long-range dependencies and multi-scale global contextual information of cardiac CT images, enabling the segmentation network to assimilate both local and global information at each feature scale, thereby achieving efficient and precise segmentation of cardiac substructures. Segmentation performance was quantitatively assessed using the Dice similarity coefficient (DSC) [10], which is commonly used metrics in medical image segmentation tasks.

#### Software system based on cardiac substructure reconstruction algorithm

Building upon the aforementioned cardiac substructure segmentation algorithm, we have further developed a software system that facilitates the process from uploading cardiac CT imaging cases to outputting STL format files compatible with 3D printing systems, comprising the following functional modules: (1) a multi-modal image import module supporting standard formats (e.g., DICOM, NIFTI) with metadata extraction; (2) an intelligent preprocessing module integrating image normalization, hybrid denoising, and multi-modal registration algorithms; (3) a cardiac substructure segmentation module employing deep learning for automated segmentation of ten anatomical structures; (4) a 3D visualization module providing multiplanar reconstruction and volume rendering capabilities; (5) a data export module supporting parameter-adjustable STL file generation; (6) a security module implementing RBAC-based access control and data security compliance with clinical privacy standards; (7) a system monitoring module with comprehensive logging and exception handling mechanisms; and (8) a user interface module featuring a clinically optimized GUI design.

### Embedded 3D printing system with silicone rubber matrix

The selection of appropriate 3D printing technology and materials is crucial for manufacturing heart models through 3D printing. These materials must be able to replicate the softness and elasticity of the heart while maintaining sufficient strength and stability during the fabrication process. Silicone rubber is considered particularly suitable for this application due to its exceptional elasticity, excellent thermal and chemical stability. Room temperature vulcanizing (RTV) silicone, in particular, offers ease of handling and can be chemically modified to achieve specific mechanical, optical, or electrical properties. These modified silicone rubbers have been widely used in fields such as soft robotics, flexible sensors, biomedical devices, and wearable technologies [23–25]. Based on these advantageous properties, we selected silicone rubber as the primary material for 3D printing cardiac substructures. The silicone rubber used in this study is an electrical insulator with very low electrical conductivity (typically on the order of 10^−12^ to 10^−15^ S/cm). This property ensures the models are electrically non-conductive, which is relevant for safety considerations in potential applications involving electrical stimulation or near electronic medical devices.

Conventional 3D printing techniques encounter substantial limitations when processing soft materials, particularly in the fabrication of complex geometric structures. These limitations include issues with material rheology, the necessity for support structures, and compromised printing precision. Embedded 3D printing technology [26] enables researchers to circumvent the limitations of traditional 3D printing techniques when processing soft materials, demonstrating great potential in the fabrication of complex structures and soft material-based products. However, the printing materials typically used in embedded 3D printing, such as silicone and hydrogels, exhibit high fluidity and moldability. The most challenging aspect of the printing process involves precise control of the support matrix’s curing parameters, particularly in terms of temporal and proportional adjustments. Silicone-based 3D printing of cardiac models faces two limitations. First, material supply is limited by reservoir capacity, typically less than 100 mL in most studies, necessitating frequent refilling during large-scale printing and continuous operator supervision to prevent print failure. Second, the printable time window is constrained, as most extrusion-based methods require pre-mixing of commercial two-part room-temperature vulcanizing (RTV) silicone, and printing must be completed before gelation. Even with curing inhibitors, the ink remains workable for only a few hours, making prolonged printing of large-volume models difficult. Furthermore, precise control of nozzle extrusion is challenging; high print speeds may lead to surface heterogeneity, compromising print quality, and most soft materials require extended curing times on the build platform.

**FIGURE 4:**
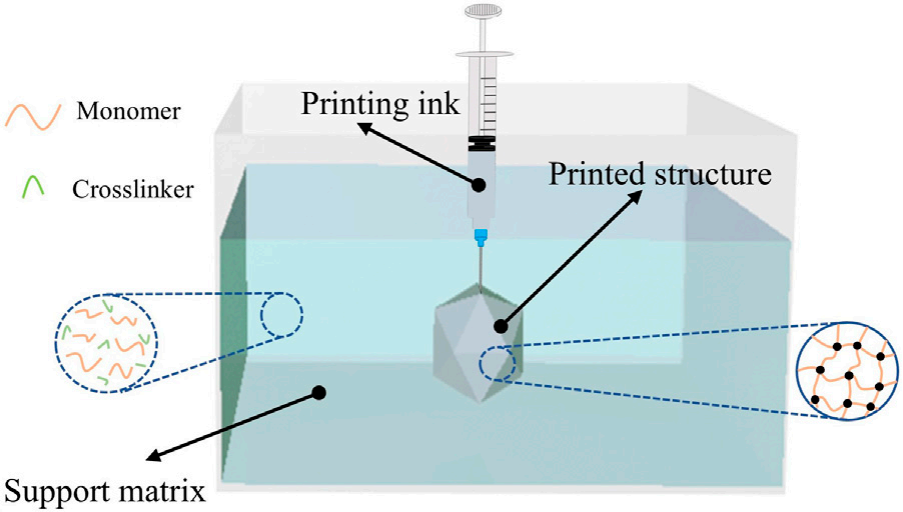
Printing principle.

To address these technical challenges, we have developed a silicone-embedded 3D printing system [27]. This system employs an advanced silicone printing methodology wherein diluted catalyst ink is continuously and uniformly extruded into a silicone polymer-containing support matrix. Through precise adjustment of the fundamental material ratios in the support matrix, we have successfully fabricated three-dimensional silicone cardiac models with complex features and high dimensional fidelity, as illustrated in the schematic diagram of the printing working principle (Figure 4) [27].

In comparison to conventional manufacturing methodologies, the advanced printing technology elucidated herein facilitates the versatile modulation of the mechanical properties of silicone rubber in response to diverse application demands. This is achieved through the precise calibration of the constituent ratios within the supporting matrix, obviating the necessity for molds or ancillary support structures. Diverging from the direct deposition of pre-cured silicone compounds, our innovative printing paradigm refines the procedural workflow by segregating the crosslinking agent from the catalyst. The methodology entails the incorporation of the crosslinking agent within the supporting matrix, whilst the catalyst is embedded within the printing ink.

**FIGURE 5:**
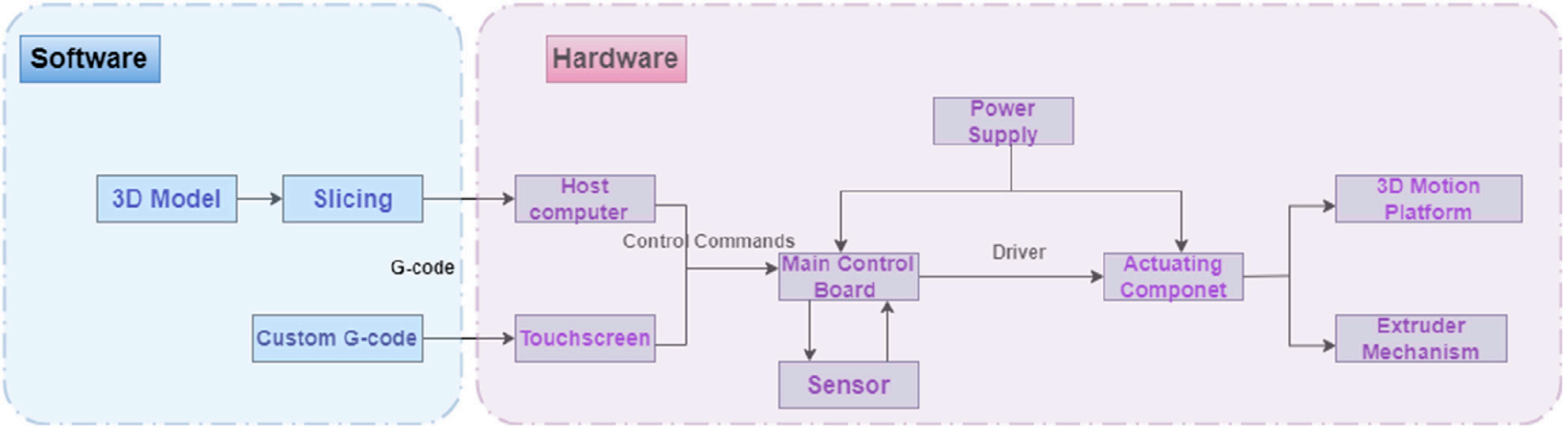
Silicone embedded 3D printing system.

During the extrusion process, the diluted catalyst-laden ink is dispensed into the supporting matrix, which is replete with the foundational silicone rubber precursors. This instigates a localized crosslinking and solidification of the adjacent matrix, thereby enabling a bespoke printing-curing sequence that augments material cohesion. Owing to the superlative catalytic efficacy, a minuscule quantity of the catalyst suffices to induce the solidification of the proximate supporting matrix. Empirical printing assays have corroborated that an ink extrusion rate of 4% engenders optimal fusion between successive printing trajectories. Consequently, the volumetric ratio of the resultant printed construct to the consumed ink approximates 25:1, indicative of an exemplary ink utilization efficiency. This innovation ameliorates the constraints imposed by finite ink reservoir capacities and protracted printing durations. The architectural schematics of the silicone-embedded 3D printing system are delineated in Figure 5.

## Experiments and results

### Experimental dataset and environment

The experimental data used in this study were provided by the Third Affiliated Hospital of Guangzhou Medical University. The dataset comprises 117 cardiac CT image samples, all acquired using a Siemens SOMATOM Force third-generation dual-source dual-energy spiral CT scanner. The constructed dataset has dimensions of 117 × 130 × 512 × 512, where 117 represents the number of cases, 130 denotes the number of slices per case, and 512 × 512 corresponds to the size of each slice. The cases were randomly divided into training, validation, and test sets in a ratio of 6:2:2.

The hardware environment for the experiments included an Intel^®^ Xeon^®^ E5-2678 v3 CPU and an NVIDIA GTX-1080Ti GPU with 11 GB of memory. The operating system was Ubuntu 18.04, and the programming language used was Python 3.7. All programs were implemented under the Pytorch open-source framework.

### Experimental results of 3D reconstruction algorithm for cardiac substructures

In this chapter, the segmentation accuracy was quantitatively evaluated using the Dice coefficient [10], which is calculated as:

$$\text{Dice}\left(X,Y\right)=\frac{2\left|G\cap P\right|}{\left|G\right|\cup \left|P\right|}$$ (1)

where G and P denote the manually segmented mask and the prediction mask, respectively, using binary tags. Dice calculates the ratio of twice the intersection of the two masks to their union, which reflects the similarity between the target region of segmentation and the annotated target region. The higher the similarity, the better the segmentation effect. Dice ranges from 0 to 1, where 1 represents the best segmentation, and 0 represents the worst segmentation.

**FIGURE 6:**
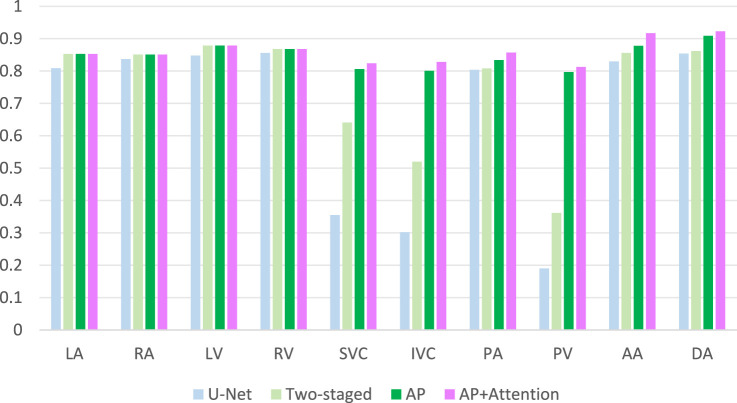
Ablation study:DSC comparison results.

To evaluate the impact of our method on segmentation performance, we compared the baseline U-Net, a two-stage U-Net, incorporating anatomical priors into a cascaded framework (AP), and our proposed framework that synergistically combines anatomical prior knowledge with a spatial-channel co-attention mechanism (AP+Attention). Results are shown in Figure 6 (The mean values calculated across all subjects in the independent test set). U-Net achieved reasonable Dice scores on large, high-contrast structures—including the four cardiac chambers (LA, RA, LV, RV) and three major arteries (AA, DA, PA)—but failed to segment small veins (SVC, IVC, PV), resulting in severe under-segmentation. The two-stage U-Net improved venous segmentation by grouping structures according to size, yielding visibly better delineation of SVC, IVC, and PV. The AP model further enhanced performance, with DSC increasing to 0.806 (SVC), 0.801 (IVC), and 0.797 (PV), demonstrating the benefit of anatomical prior integration in improving shape consistency and boundary accuracy. Our proposed AP+Attention framework achieved the highest DSC across all substructures. It showed the most significant gains in the most challenging small vessels—SVC (0.824), IVC (0.828), PV (0.813)—and attained state-of-the-art performance on the aorta (AA: 0.917, DA: 0.923). These results highlight the effectiveness of attention mechanisms in refining feature representation and enhancing segmentation accuracy, particularly for fine-grained cardiac structures.

**FIGURE 7:**
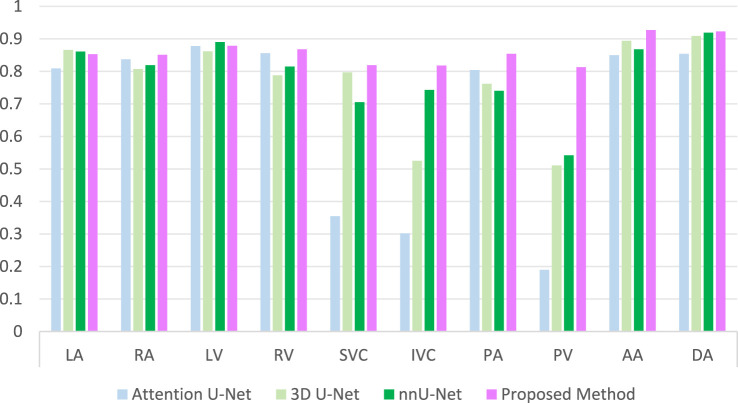
Comparison of DSC results with other methods.

To validate the performance of the proposed method, we conducted a comparative analysis with several prominent deep learning methods in the field of segmentation, including Attention U-Net [28], 3D U-Net [29], and nnU-Net [30]. The comparison is graphically illustrated in Figure 7. As evident from the comparative outcomes depicted in the figures, our method yielded modest yet consistent improvements in the traditional segmentation of cardiac chambers, specifically in RA, LV, LA and RV. Notably, our approach demonstrated a significant advantage in the segmentation of PA, DA and AA. Most strikingly, our method excelled in the challenging categories of SVC, IVC and PV, which are characterized by small sizes and low sample counts. Particularly for PV, neither 3D U-Net nor nnU-Net achieved a Dice score exceeding 0.6, indicating inadequate segmentation. This highlights the superior capability of our method to achieve accurate and comprehensive segmentation of these structures.

### Experimental design for embedded 3D printing

**FIGURE 8:**
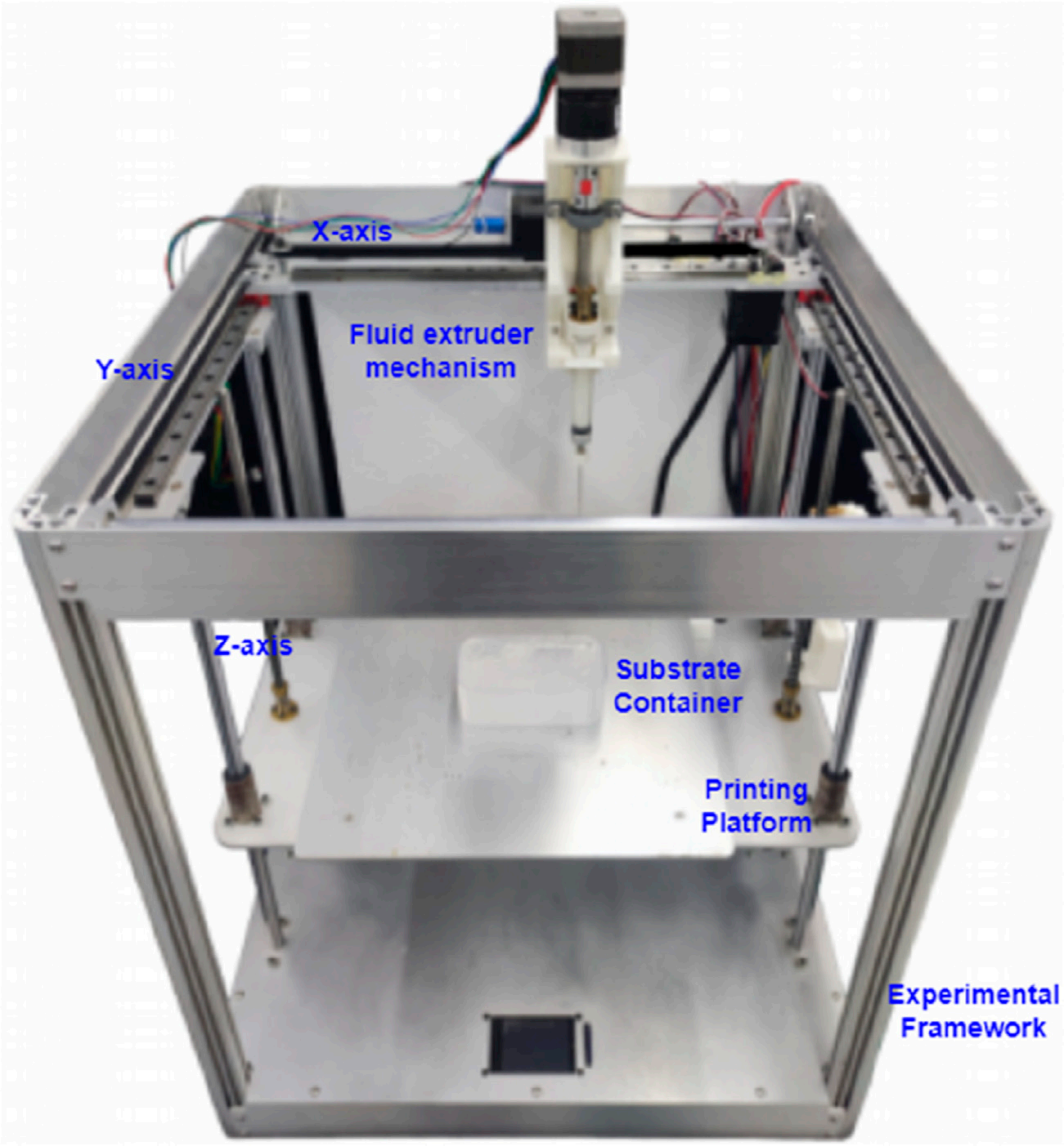
Silicone embedded 3D printing experimental platform.

The experimental platform for this study is illustrated in Figure 8, which is an XYZ frame 3D printing system using silicone as the support matrix and equipped with a motor-lead screw fluid extrusion mechanism.

**FIGURE 9:**
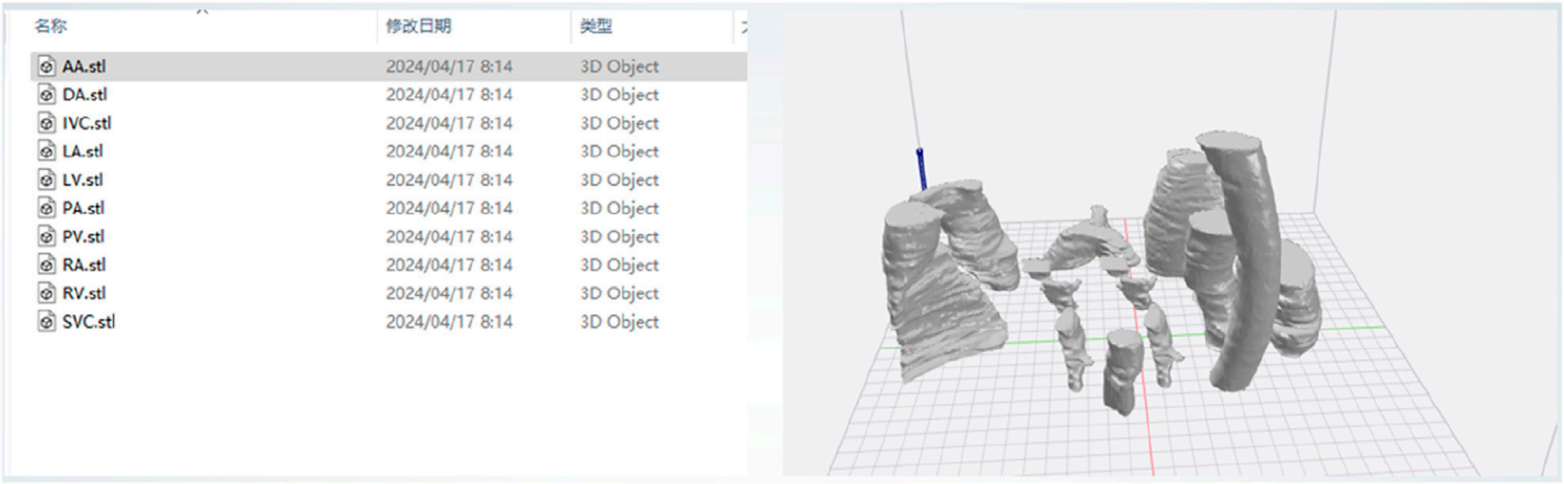
3D model in STL format.

**FIGURE 10:**
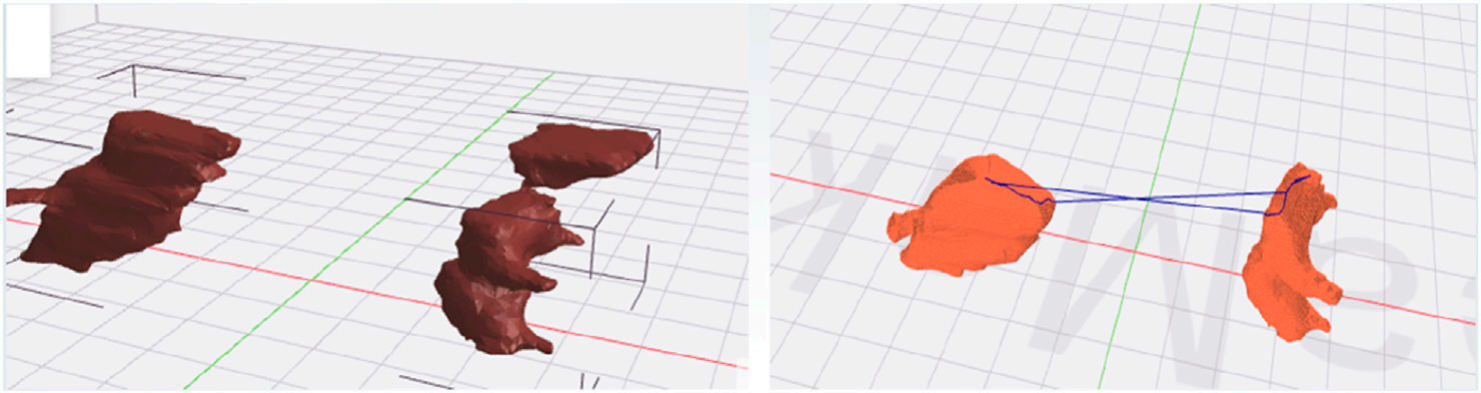
Conversion of STL format model to G-Code format model.

The specific steps of the printing process are as follows:

1. Export the 3D model in STL format from the software system based on the heart substructure reconstruction algorithm described in Section *Software system based on cardiac substructure reconstruction algorithm* (as shown in Figure 9).
2. Use slicing software such as Ultimaker Cura or IdeaMaker to convert the STL format model into G-code, which the 3D printing system can understand and execute, and then send it to the 3D printing system (as shown in Figure 10).

G-code is a crucial part of the printing system, as it directly determines the structure and quality of the printed object. The designed 3D model is imported into the slicing software in STL format. First, the position, size, and orientation of the model are adjusted to achieve the optimal printing direction. The slicing software then layers the model based on the set printing parameters and uses built-in slicing algorithms to plan the printing path and control the speed for each layer, ultimately generating the G-code instruction file. The printer interprets these instructions and constructs the 3D object layer by layer along a specific path and speed.

The slicing software contains numerous parameters, with the most critical printing parameters including printing speed, layer height, line width, extrusion rate, infill density, and infill pattern. Only a combination of printing parameters that match the material and structural characteristics can achieve the best printing quality. For different printing materials and processes, parameter optimization experiments are necessary to improve printing quality. Through experimental testing, the printing parameters for this study are set as follows: layer height 0.4mm, line width 0.3mm, infill density 100%, extrusion rate 4%, printing speed 15 mm/s, and infill pattern: concentric circles.

After processing the model with the slicing software to obtain the G-code for layer-by-layer printing, additional lines of G-code are added before and after the start and end of the G-code generated by the slicing software. These lines control the lifting of the printing platform, aiming to lower the platform to a position where the needle tip does not overlap with the matrix container in the Z-axis direction. This prevents the needle from colliding with the container during the homing process, which could damage the system.

3. Preparation of Printing Materials: Prepare the support matrix and printing ink.

The main materials used for the support matrix include high-viscosity vinyl silicone oil MP5000, hydrogen-containing silicone oil MH180, hydrogen-containing silicone oil MDH50, and fumed silica A380. Among these, MP5000 and MH180 are the primary reactants, MDH50 adjusts the crosslinking density of the network to modify the mechanical properties of the printed silicone rubber material, and A380 acts as a rheological modifier to regulate the rheological properties of the support matrix. The printing ink is a platinum catalyst diluted with vinyl silicone oil MP450. Additionally, to enhance the visualization of the printing paths, a small amount of color paste or fluorescent powder in different colors is mixed into the ink for different substructures of the heart.

The support matrix is poured into a container, degassed, and then placed on the printing platform. The printing ink is loaded into the reservoir of the material supply system, ready for printing.

**FIGURE 11:**
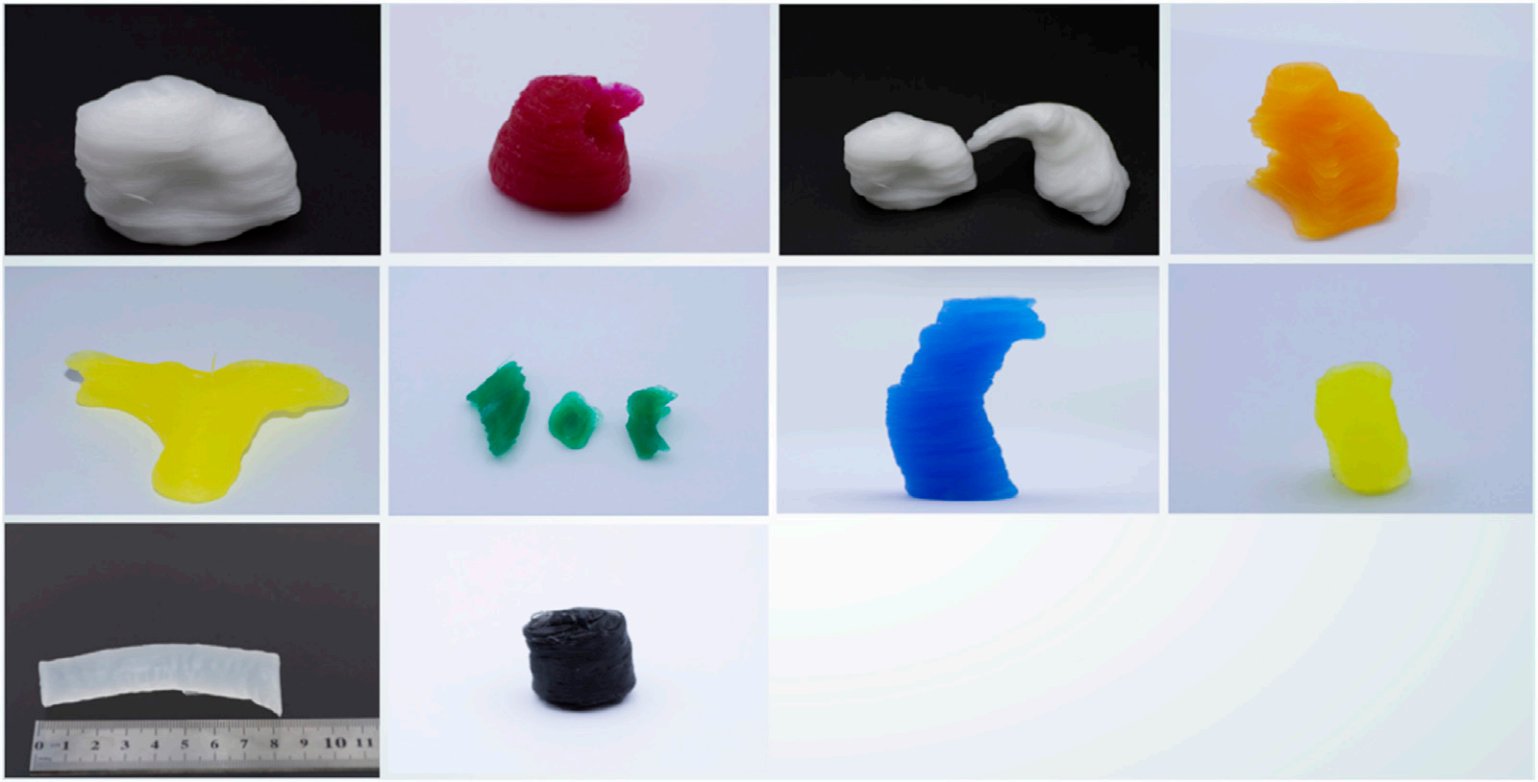
3D models of printed cardiac substructures.

4. After all materials have been printed, heat is applied to accelerate curing. The printed structures are placed in a heating chamber at 70 °C to speed up the curing process, and they are removed after approximately 2 h. The printing results are shown in Figure 11.

### 3D printing results

**FIGURE 12:**
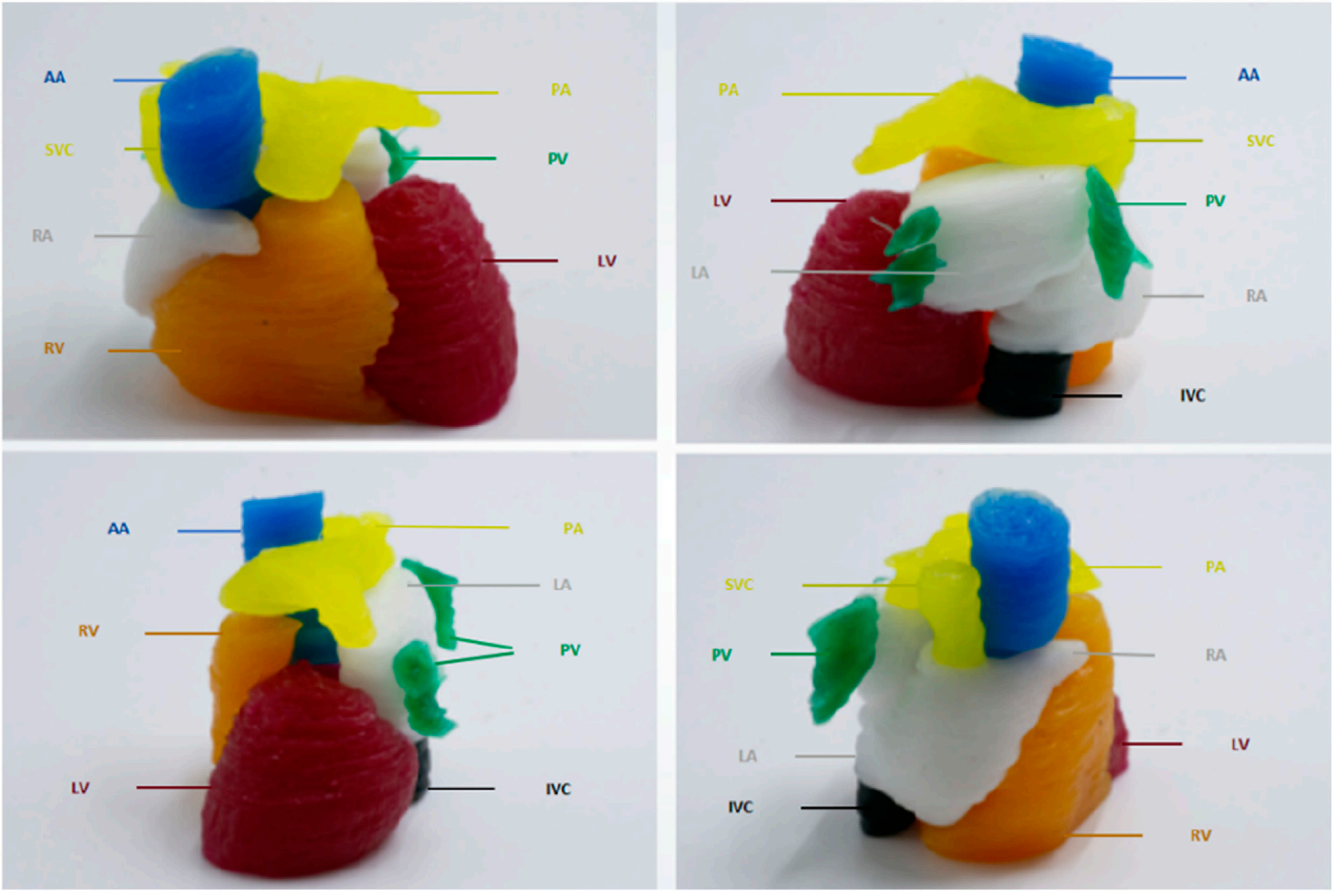
3D models of cardiac substructures.

**FIGURE 13:**
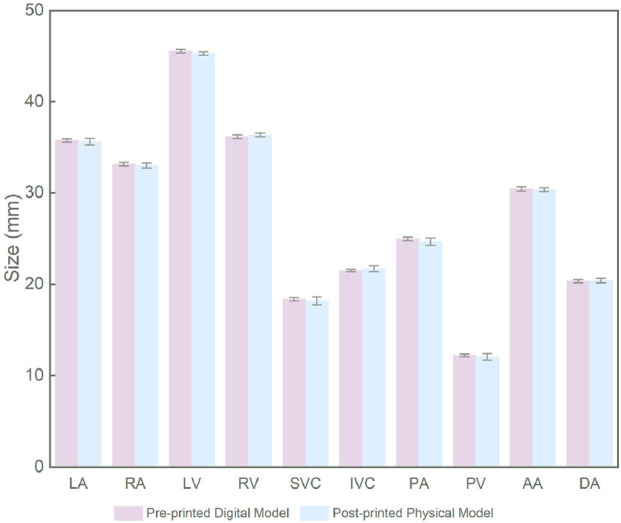
Comparison of pre-printed and post-printed models.

The printed cardiac substructures were assembled by a radiologist using a minimal amount of adhesive (silicone adhesive J-527S) to precisely align and bond the components, ensuring tight junctions without significant material buildup. The adhesive cures rapidly at room temperature, forming a strong and durable connection. Anatomical landmarks were carefully referenced during alignment to maintain spatial relationships and overall morphological accuracy. The 3D heart model is shown in Figure 12. The measurement results of the pre-printed digital model and post-printed physical model are presented in Table 1, with comparative data illustrated in Figure 13. The data were derived from a single, representative patient-specific 3D-printed cardiac model. To assess fidelity, each anatomical landmark was measured by the physicians. The values reported are the means of these measurements, with the ± indicating the standard deviation (SD), reflecting variability due to measurement technique and minor surface irregularities. All SDs for the physical measurements were less than 0.5 mm, indicating measurement consistency, while the mean absolute differences (MADs) ranging from 0.15 to 0.46 mm demonstrate dimensional accuracy within clinically acceptable limits.

**TABLE 1:** The measurement results of the digital model before printing and the physical model after printing.

Substructures	Measurement	Pre-printed digital model (mm)	Post-printed physical model (mm)	Mean absolute difference (mm)
LA	The maximum transverse diameter, measured at the level of the middle of the atrium, perpendicular to the interatrial septum	35.75 ± 0.22	35.41 ± 0.37	0.35
RA	The maximum transverse diameter, measured at the level of the middle of the atrium, from the lateral wall to the interatrial septum	33.15 ± 0.18	33.0 ± 0.26	0.15
LV	The short-axis diameter, measured at the level of the papillary muscles in the short-axis view, from the interventricular septum to the lateral wall	45.63 ± 0.22	45.21 ± 0.20	0.42
RV	The short-axis diameter, measured at the basal short-axis view, from the free wall to the interventricular septum	36.15 ± 0.19	36.38 ± 0.18	0.23
SVC	Diameter, measured at a transverse Section *Introduction* cm above the entrance of the right atrium	18.38 ± 0.18	17.92 ± 0.45	0.46
IVC	Diameter, measured at the transverse section at the level of the diaphragm	21.5 ± 0.14	21.78 ± 0.32	0.28
PA	Diameter, measured at a transverse Section *Introduction* cm above the valve level	24.95 ± 0.19	24.54 ± 0.40	0.41
PV	Diameter, measured at the transverse section at the junction of the left atrium (at the thickest point of the left superior pulmonary vein)	12.2 ± 0.14	11.82 ± 0.37	0.38
AA	Diameter, measured perpendicular to the direction of blood flow	30.48 ± 0.22	30.32 ± 0.21	0.16
DA	Diameter, measured at the transverse section above the diaphragm	20.33 ± 0.19	20.5 ± 0.24	0.17

## Discussion

In this study, we developed a comprehensive 3D reconstruction framework for cardiac substructures, enabling the precise replication of patient-specific cardiac anatomy and the creation of highly personalized heart models. This framework integrates two subsystems: a 3D reconstruction system for cardiac substructures and an embedded 3D printing system utilizing a silicone rubber matrix. The workflow encompasses the entire process, from uploading cardiac CT imaging data to generating STL files compatible with 3D printing systems, followed by embedded 3D printing with silicone rubber. This approach achieves automated and intelligent 3D reconstruction of cardiac substructures, addressing two critical challenges in the field: (1) enhancing data accuracy during the pre-processing phase through a novel cardiac substructure reconstruction algorithm that incorporates anatomical prior knowledge and a collaborative spatial-channel attention mechanism, and (2) enabling high-fidelity fabrication of complex 3D silicone heart models without the need for molds or support structures by optimizing the composition of the support matrix.

Accurate segmentation of cardiac substructures in CT remains challenging due to large variations in size, shape, intensity, and spatial location—e.g., the left ventricle is approximately ten times larger in volume than the superior vena cava. Conventional end-to-end models underperform on small, low-contrast structures like the SVC, IVC, and PV, especially with limited training data. To address this, we propose a two-stage framework integrating anatomical priors with a spatial-channel co-attention mechanism. The first stage segments large substructures (e.g., ventricles, atria), whose outputs are fused with the original image to guide the second-stage refinement network. This grouped strategy improves small-structure accuracy, increasing Dice scores for SVC, IVC, and PV by 20%–30%. By embedding spatial, morphological, and scale priors from larger substructures, boundary delineation and shape consistency are enhanced, raising DSC to 0.806 (SVC), 0.801 (IVC), and 0.797 (PV). A spatial-channel collaborative attention module at the encoder-decoder interface further improves feature discrimination by fusing channel and spatial attention, strengthening global-local fusion and suppressing background noise—critical for resolving small, blurred vessels. As shown in Figure 6, the full model achieves peak performance on the most challenging targets: DSC = 0.824 (SVC), 0.828 (IVC), 0.813 (PV), and 0.917/0.923 (AA/DA). Compared to mainstream models (Figure 7), our method elevates Dice scores for small substructures from <0.35 to >0.82, demonstrating accuracy in handling scale disparity and low contrast for comprehensive cardiac segmentation.

Material selection is critical for achieving biomimetic fidelity in 3D-printed cardiac models. In this study, we selected silicone rubber as the primary printing material due to its superior mechanical properties and biocompatibility, enabling realistic cardiac simulation. Compared to common alternatives, silicone offers distinct advantages: thermoplastics such as polylactic acid (PLA) are accessible and suitable for rapid prototyping of basic anatomical structures [31], but lack the softness; thermoplastic polyurethane (TPU) improves flexibility for soft-tissue simulation [32], yet its mechanical behavior may deviate from the nonlinear elasticity of heart muscle; photopolymer resins enable high-resolution printing with excellent surface finish, making them ideal for detailed anatomical models in preoperative planning and education [33], although many commercial formulations do not meet medical-grade standards; while cell-laden bioinks hold promise for tissue engineering [34], they remain challenging for fabricating structurally robust and dimensionally stable models for surgical training. In contrast, silicone exhibits exceptional elasticity, tunable mechanical properties, and high thermal and chemical stability, closely mimicking the viscoelastic behavior of native cardiac tissue, with proven safety for clinical handling and repeated use [35]. Furthermore, advances in embedded 3D printing enable the precise fabrication of complex, multi-chambered cardiac models with silicone, overcoming limitations of traditional molding or layer-based rigid printing.

Silicone-based cardiac printing faces two limitations: (1) limited reservoir capacity (typically <100 mL), necessitating frequent material refilling and continuous supervision to prevent print failure during large-scale fabrication; and (2) constrained working time, as most extrusion-based methods require pre-mixing two-part RTV silicone, with printing completed before gelation. Even with inhibitors, ink usability lasts only a few hours, limiting prolonged printing of large constructs. This study presents an advanced embedded 3D printing method in which a diluted catalytic ink is extruded into a silicone polymer-laden support matrix. This approach enables mold-free, support-free fabrication of complex 3D cardiac models with high fidelity. By tuning the composition of the support matrix, the mechanical properties of the printed silicone can be precisely tailored to specific applications. Unlike conventional methods that deposit pre-mixed silicone, our system decouples the crosslinking agent (in the matrix) from the catalyst (in the ink). During printing, localized curing is triggered upon ink deposition, enabling on-demand, layer-by-layer solidification. This strategy enhances material integration and overcomes limitations in ink reservoir capacity and working time, facilitating extended, uninterrupted printing of large-scale, anatomically accurate cardiac structures.

This study has several limitations. First, although the segmentation algorithm integrating anatomical priors and attention fusion demonstrates promising performance, its accuracy for small or low-contrast structures could be further improved; future work will explore advanced techniques to enhance segmentation of challenging substructures. Second, we plan to evaluate the adaptability of our method to other biomedical imaging modalities, such as MRI and ultrasound, to assess its robustness and generalizability. Despite these limitations, our integrated framework holds significant potential for personalized clinical applications. The cardiac models enable preoperative planning for heart diseases, allowing surgeons to visualize anatomical relationships and simulate procedures, while also serving as an auxiliary platform for surgical training. The modular design facilitates extension to biomimetic modeling of other soft tissues—such as liver and kidney—by adapting the algorithm and tuning material mechanics, with applications in oncologic surgery planning. Future efforts will focus on incorporating micro-sensors for dynamic functional simulation, developing biodegradable or bioactive silicones, and automating the entire pipeline to reduce turnaround time and enhance clinical translation, ultimately supporting surgical innovation, patient-physician communication and medical education.

## Conclusion

To address limitations in manufacturing complexity, geometric fidelity, and personalization of existing cardiac models, this study presents an innovative approach integrating artificial intelligence (AI) and embedded 3D printing for reconstructing patient-specific cardiac substructures. The proposed framework leverages medical imaging data (e.g., CT scans) and advanced 3D reconstruction algorithms to automatically segment and model cardiac anatomy, significantly enhancing image resolution and accuracy during preprocessing and generating high-quality 3D printable files. Furthermore, an embedded 3D printing technique based on a silicone rubber matrix enables on-demand printing and curing through precise modulation of the support matrix composition, facilitating the fabrication of highly complex, high-fidelity silicone heart models. These models accurately replicate patient-specific cardiac anatomy, providing valuable morphological insights for diagnosis and treatment. The framework shows strong potential for personalized preoperative planning and training, with a modular design adaptable to other soft tissues for broader biomedical applications. Future efforts will focus on improving printing precision and efficiency to enhance clinical translation, ultimately supporting surgical innovation, patient-physician communication, and medical education.

## Data Availability

The data analyzed in this study is subject to the following licenses/restrictions: The datasets generated during this study are property of the participating hospital and are not publicly available due to institutional data governance policies. Data access requires approval from the hospital ethics committee. Requests to access these datasets should be directed to TS flair@gzhmu.edu.cn.

## References

[1] World Heart Federation (WHF)[EB/OL]. World heart Federation. Available online at: https://world-heart-federation.org/ (Accessed September 17, 2025).

[2] Payer C Štern D Bischof H. Multilabel whole heart segmentation using cnns and anatomical label configurations. In: International workshop on statistical atlases and computational models of the heart. Springer (2017).190198

[3] Wang C Smedby Ö. Automatic whole heart segmentation using deep learning and shape context. In: International workshop on statistical atlases and computational models of the heart. Springer (2017).242249

[4] Yang X Bian C Yu L. 3d convolutional networks for fully automatic finegrained whole heart partition. In: International workshop on statistical atlases and computational models of the heart. Springer (2017).181189

[5] Savaashe AK Dharwadkar NV. A review on cardiac image segmentation. In: 2019 3rd international conference on computing methodologies and communication (ICCMC). India: Erode (2019). p. 545–50.

[6] Zhuang X. Evaluation of algorithms for multi-modality whole heart segmentation: an open-access grand challenge. Med Image Anal (2019) 55:92–102. 10.1016/j.media.2019.101537 31446280 PMC6839613

[7] Yang X Zhang Y Lo B Wu D Liao H Zhang Y -T. DBAN: adversarial network with multi-scale features for cardiac MRI segmentation. IEEE J Biomed Health Inform (2021) 25(6):2018–28. 10.1109/jbhi.2020.3028463 33006934

[8] Slobodzian V Radiuk P Barmak O Krak I. Multi-stage segmentation and cascade classification methods for improving cardiac MRI analysis. J Med Imaging Health Inform (2023) 13(4):567–78. 10.48550/arXiv.2412.09386

[9] Bui V Hsu L-Y Chang L-C Sun A-Y Tran L Shanbhag SM DeepHeartCT: a fully automatic artificial intelligence hybrid framework based on convolutional neural network and multi-atlas segmentation for multi-structure cardiac computed tomography angiography image segmentation. J Med Imaging Health Inform (2023) 13(5):789–801. 10.3389/frai.2022.1059007 36483981 PMC9723331

[10] Ronneberger O Fischer P Brox T. U-Net: Convolutional networks for biomedical image segmentation. Lecture Notes Computer Sci (2015) 234–41. 10.1007/978-3-319-24574-4_28

[11] Diniz JOB Dias Júnior DA da Cruz LB da Silva GLF Ferreira JL Pontes DBQ Heart segmentation in planning CT using 2.5D U-Net++ with attention gate. Computer Methods Biomech Biomed Eng Imaging & Visualization (2023) 11(3):317–25. 10.1080/21681163.2022.2043779

[12] Qiao J Wang X Chen J Liu M. MBUTransNet: multi-branch U-shaped network fusion transformer architecture for medical image segmentation. Int J Computer Assisted Radiol Surg (2023) 18(10):1895–902. 10.1007/s11548-023-02879-1 37024630

[13] Jégou S Drozdzal M Vazquez D Romero A Bengio Y. The one hundred layers tiramisu: fully convolutional densenets for semantic segmentation. In: 2017 IEEE conference on computer vision and pattern recognition workshops (CVPRW) (2017). p. 1175–83.

[14] Roth HR Lu L Farag A Shin H-C Liu J Turkbey EB DeepOrgan: multi-level deep convolutional networks for automated pancreas segmentation. In: International conference on medical image computing and computer-assisted intervention (MICCAI) (2015). p. 556–64.

[15] Zhao Y Wang X Che T Bao G Li S. Multi-task deep learning for medical image computing and analysis: A review. Comput Biol Med (2023) 153: 106496. 10.1016/j.compbiomed.2022.106496 36634599

[16] Singh KR Sharma A Singh GK. MADRU-Net: multiscale attention-based cardiac MRI segmentation using deep residual U-Net. IEEE Trans Instrum Meas (2024) 73: 1–13. 10.1109/TIM.2023.3332340

[17] Song Y Lu Y Fu X Wong KKL. Deep learning-based automatic segmentation of images in cardiac radiography: a promising challenge. Comput Methods Programs Biomed (2022) 220: 106821. 10.1016/j.cmpb.2022.106821 35487181

[18] Gharleghi R Dessalles CA Lal R McCraith S Sarathy K Jepson N 3D printing for cardiovascular applications: from end-to-end processes to emerging developments. Ann Biomed Eng (2021) 49: 1598–1618. 10.1007/s10439-021-02784-1 34002286 PMC8648709

[19] Valverde I. Three-dimensional printed cardiac models: applications in the field of medical education, cardiovascular surgery, and structural heart interventions. Revista Española de Cardiología (English Edition) (2017) 70(4):282–91. 10.1016/j.rec.2017.01.012 28189544

[20] Noor N Shapira A Edri R Gal I Wertheim L Dvir T. 3D printing of personalized thick and perfusable cardiac patches and hearts. Adv Sci (2019) 6(11):1900344. 10.1002/advs.201900344 31179230 PMC6548966

[21] Sun Z Wee C. 3D printed models in cardiovascular disease: an exciting future to deliver personalized medicine. Micromachines (Basel). (2022) 13(10):1575. 10.3390/mi13101575 36295929 PMC9610217

[22] Wang X Li X Du R Zhong Y Lu Y Song T. Anatomical prior-based automatic segmentation for cardiac substructures from computed tomography images. Bioengineering (2023) 10:1267–85. 10.3390/bioengineering10111267 38002391 PMC10669053

[23] Truby RL Lewis JA. Printing soft matter in three dimensions. Nature (2016) 540(7633):371–8. 10.1038/nature21003 27974748

[24] Yuk H Lin S Ma C Takaffoli M Fang NX Zhao X. Hydraulic hydrogel actuators and robots optically and sonically camouflaged in water. Nat Commun (2017) 8(1):14230. 10.1038/ncomms14230 28145412 PMC5296644

[25] Wehner M Truby RL Fitzgerald DJ Mosadegh B Whitesides GM Lewis JA An integrated design and fabrication strategy for entirely soft, autonomous robots. Nature (2016) 536(7617):451–5. 10.1038/nature19100 27558065

[26] Brown AL Gaston AP Truby RL Lewis JA. Embedded 3D printing for complex geometry and soft materials. Adv Mater Tech (2018). 10.1038/nature19100

[27] Li Y Wu Z Chen Y Xian S Hong Z Wang Q Multi-material embedded 3D printing for one-step manufacturing of multifunctional components in soft robotics. Additive Manufacturing (2024) 85:104178. 10.1016/j.addma.2024.104178

[28] Oktay O. Attention U-Net: learning where to look for the pancreas. arXiv preprint arXiv:1804.03999 (2018).

[29] Ciçek Ö Abdulkadir A Lienkamp SS. 3D U-net: learning dense volumetric segmentation from sparse annotation. In: International conference on medical image computing and computerassisted intervention (2016). p. 424–32.

[30] Isensee F Jaeger PF Kohl SAA Petersen J Maier-Hein KH. nnU-Net: a self-configuring method for deep learning-based biomedical image segmentation. Nat Methods (2021) 18:203–11. 10.1038/s41592-020-01008-z 33288961

[31] Wei J Oyunbaatar N-E Jeong Y-J Park J Kim S-H Kwon K Enhancing flexibility of smart bioresorbable vascular scaffolds through 3D printing using polycaprolactone and polylactic acid. Sensors Actuators B: Chem (2025) 422:136667. 10.1016/j.snb.2024.136667

[32] Gasparotti E Vignali E Losi P Scatto M Fanni BM Soldani G A 3D printed melt-compounded antibiotic loaded thermoplastic polyurethane heart valve ring design: an integrated framework of experimental material tests and numerical simulations. Int J Polymeric Mater Polymeric Biomater (2018) 68(1–3):1–10. 10.1080/00914037.2018.1525717

[33] Ertas A Farley-Talamantes E Cuvalci O Gecgel O. 3D-Printing of artificial aortic heart valve using UV-Cured silicone: design and performance analysis. Bioengineering (2025) 12(1):94. 10.3390/bioengineering12010094 39851368 PMC11761925

[34] Zhu K Shin SR van Kempen T Li Y Ponraj V Nasajpour A Gold nanocomposite bioink for printing 3D cardiac constructs. Adv Funct Mater (2017) 27(12):1605352. 10.1002/adfm.201605352 30319321 PMC6181228

[35] Dehnou KH Hadianfard MJ. Enhancing thermal, viscoelastic, and mechanical properties of silicone rubber matrix through reinforcements for use as a medical implant. Recent Prog Mater (2024) 6(2):011. 10.21926/rpm.2402011

